# *Fomes fomentarius* and *F. inzengae*—A Comparison of Their Decay Patterns on Beech Wood

**DOI:** 10.3390/microorganisms11030679

**Published:** 2023-03-07

**Authors:** Valentino Cristini, Patrik Nop, Jan Zlámal, Mojtaba Hassan Vand, Vít Šeda, Jan Tippner

**Affiliations:** Department of Wood Science and Technology, Mendel University in Brno, 613 00 Brno, Czech Republic

**Keywords:** biodegradation, nondestructive testing, tree biomechanics, tree stability, white rot, wood-decaying fungi

## Abstract

Wood-decaying fungi are responsible for the degradation of wood and the alteration in its material properties. *Fomes fomentarius* (L.) Fr. is one of the most common white-rot fungi colonising coarse wood and standing trees. In recent years, according to their genetic, physiological, and morphological differences, *Fomes inzengae* (Ces. and De Not.) Lécuru was identified as an independent species. This article aimed to compare the impact of the degradation caused by both species on the anatomical, physical, and mechanical properties of beech wood. When comparing the degradation caused by different strains of both species, no statistically significant difference was found in mass loss (ML) or moisture content (MC). A relevant correlation between ML and MC was confirmed for both species. Variabilities in the density distribution of the degraded and intact bending samples were found to be statistically different. No relevant difference was observed in the modulus of rupture (MOR) between the two species after each exposure period. A strong linear relationship between the MOR and the dynamic modulus of elasticity was revealed for both species. Both species showed decay patterns typical for simultaneous white rot and soft rot. According to the presented results, the impact of both species on the investigated material properties of wood cannot be considered significantly different.

## 1. Introduction

Wood-decaying fungi, through alterations in the physicochemical properties of wood, can lead to a high ecological potential of dead wood and colonised standing trees. This can contribute to the preservation of different protected species and promote higher biodiversity [1,2]. Considering all the anthropogenic factors influencing the ecological service of trees [3], the importance of specific microhabitats on mature specimens colonised by wood-decaying fungi is rising [4]. Nevertheless, stressed trees are more inclined to different negative abiotic and biotic factors [5], which can irreversibly compromise the host tree’s stability [6]. To prevent such events, the extent of decay and tree stability are often evaluated through nondestructive device-supported methods [7,8]. One of the most common wood-decaying fungi influencing trees’ structural stability is *F. fomentarius* [6,9] whose presence is widely spread in both forest and urban environments [10,11]. *F*. *fomentarius* is a simultaneous white-rot fungus that can be found as one of the main decomposers of the coarse wood of different species such as *Fagus sylvatica* L. (*F. sylvatica*), *Tilia cordata* Mill. or *Betula pendula* Roth. [12,13,14]. On the other hand, *F. fomentarius* is known as a heart-rot fungus in standing trees, often starting its degradation after previous colonisation through mechanical wounds or dead stubs and acting as an endophyte for many years [15,16]. Nevertheless, in comparison to other species with an endophytic strategy, *F. fomentarius* has a more combative behaviour [17]. Peintner et al. [18] proposed *F. inzengae*, previously considered just a synonym of *F. fomentarius*, as a distinct species. Besides the similarity of their basidiomes, *F. inzengae* differs from *F. fomentarius* in many micro-morphological, physiological, and genetic characteristics. Badalyan et al. [19], according to the results obtained by the internal transcribed spacer (ITS) DNA sequence barcoding of the two species, suggested that *F. fomentarius sensu stricto* (*s.s.*) and *F. inzengae* are possibly not taxonomically separate species but sympatric cryptic subspecies of *F. fomentarius sensu lato*. Considering the novelty of this topic, further investigation concerning the differences in the decay behaviour between the two species is necessary. Furthermore, since tree stability is a parameter usually assessed visually [20], detailed knowledge of this decay behaviour and its consequent impact on the mechanical properties of colonised wood could improve contemporary evaluation procedures. The knowledge of any relevant difference in their decay behaviour and their impact on the mechanical properties of colonised wood can have an important impact on the field of visual tree stability assessment. Mass loss (ML) is a common parameter used to assess the impact of fungal degradation on wood, as well as between different fungal species [21,22,23]. Besides mass-loss measurement, the properties of degraded wood can also be assessed through nondestructive vibroacoustic techniques [24], which can lead to a better prediction even during incipient decay [25]. The impact of the colonisation of wood-decaying fungi in standing trees on the mechanical and physical properties of wood has been already tested in the past: Schwarze et al. [26] investigated the decay patterns of the white-rot fungus *Inonotus hispidus* (Bull.) P. Karst. on ash and London plane wood, proving that in certain situations, the fungus can switch to a different decay pattern typical for soft-rot fungi. Deflorio et al. [27] studied the decay development of six different fungal species in the standing trees of four different wood species, revealing different results for nondestructive testing and ML (after tree felling) depending on the time of inoculation. Cristini et al. [28] tested the physical and dynamic/static bending mechanical properties of beech wood artificially inoculated with the soft-rot fungus *Kretzschmaria deusta* (Hoffm.) P.M.D. Martin, indicating higher heterogeneity of density among singular samples due to fungal degradation and a strong correlation between the modulus of rupture (MOR) and the dynamic modulus of elasticity (MOED). The available data on *F. fomentarius* are scant, with some reports mainly focusing on competitive abilities against other wood-decaying fungi on agar soil [29] growth rate and ML on spruce and birch wood [14], and degradation abilities when growing on beech wood powder [30]. Studies concerning the impact of the degradation of *F. fomentarius* on the material properties of colonised wood are still missing. This article aims to compare the impact of the degradation of *F. fomentarius s.s.* and *F. inzengae* on the morphological, physical, and dynamic/static mechanical properties of beech wood. A deeper understanding of their interspecific differences can lead to a better interpretation of their presence on standing trees during visual tree assessment.

## 2. Materials and Methods

### 2.1. Sample Preparation

**Figure 1:**
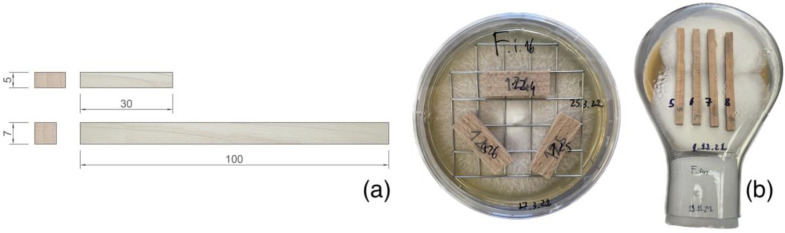
Front and lateral faces of crafted specimens for mass-loss assessment and mechanical testing (**a**); fungal exposure in Petri dishes and Kolle culture flasks (**b**).

All the samples of European beech (*Fagus sylvatica* L.) were crafted in March 2022 from fresh wood boards. The chosen wood came from the University Forest Enterprise Masarykův les Křtiny (Czech Republic). For the samples’ crafting, only intact uncolonised sapwood was used. For this article, two main kinds of samples were crafted. For the mass-loss test, small beech wood samples were cut 10 × 5 × 30 mm according to the method developed by Bravery [31]. For mechanical testing, small orthotropic bending samples (7 × 7 × 100 mm) of European beech (*F. sylvatica*) were crafted (Figure 1a). These specimens were cut according to the methodology presented by Cristini et al. [28]. To obtain oven-dry mass, the samples were completely dried in a kiln for two days at 103 °C and weighed after approaching steady conditions. Before vibroacoustic measurement and the samples’ inoculation, a higher moisture content (MC= 60 ± 10%) was established through vacuum impregnation with demineralised water (20 kPa, 1–3 min).

The moisture content (MC), green density ($\rho_{w}$), basic density ($\rho_{c}$), and ML were calculated from sample dimensions and both dry and wet (before and after degradation) masses.

### 2.2. Fungal Exposure

#### 2.2.1. Selection of Fungal Strains

For the fungal exposure experiment, five strains of each species *F. fomentarius s.s*. (referred to as just *F. fomentarius*) and *F. inzengae* were selected (Table 1). Mechanical samples were exposed to the strains (one for each species) with ML values close to the average values obtained from all the tested strains, which were isolated from basidiomes growing on beech trees. The 2 mm piece of mycelium of the freshly grown culture was put into 20 μL of dilution buffer (a component of Phire Plant Direct PCR Master Mix, Thermo Fisher Scientific). The solution was used as a template for a PCR reaction using primer pair ITS1/ITS4 targeting the ITS region of the ribosomal RNA gene during the standard procedure [32]. The strains were identified using ITS DNA sequence barcoding, with the data presented by Peintner [18] serving as a reference. The used strains were also selected according to their growth rates. All the strains were isolated and identified at the Mycological Laboratory of the Department of Forest Protection and Wildlife Management, Mendel University in Brno.

#### 2.2.2. Sterilisation Process

Prior to fungal exposure, all the specimens were sterilised by steam for 10 min at 120 °C after being exposed for 24 h at 103 °C to obtain dry, intact masses. This modified approach was adopted to achieve effective sterilisation without altering the mechanical properties of the wood by exposing it to high temperatures for longer periods. The sterilisation process was tested before starting the experiment using 20 bending specimens measuring 7 × 7 × 100 mm. The samples were stored in glass Petri dishes for 4 weeks under the same conditions as they would be exposed to during fungal exposure. No contamination or ML was detected in these samples.

#### 2.2.3. Mass-Loss Assessment

After sterilisation, small wooden samples for ML were exposed to fungal activity in Petri dishes. Each Petri dish was previously filled with 20 mL of sterile malt extract agar medium (5%) and inoculated with all the selected fungal strains. Prior to inoculation, mycelium was cultivated on small beech wood blocks for 4 weeks to stimulate enzymatic activity. A total of 120 Petri dishes were prepared, with 12 dishes for each fungal strain. Following the international standard CEN/TS 15083 [33] and the method by Bravery [31], no controls were used for the exposition process. Each Petri dish contained three samples exposed to fungal degradation (Figure 1b). Every 2 weeks after inoculation (i.e., at 2, 4, 6, and 8 weeks), 9 samples (3 Petri dishes) for each strain were taken from the dishes. The samples were cleaned of superficial mycelium and dried in a kiln for two days at 103 °C, and their mass was measured under steady conditions. Mass loss (ML) was then calculated as a percentage of the dry mass lost during the exposure period.

#### 2.2.4. Fungal Exposure of Samples for Mechanical Testing

Kolle culture flasks (400 mL) containing 70 mL of sterile malt extract agar medium (5%) were used for fungal exposure of the bending samples. After two weeks of incubation at 22 °C and 70% relative humidity, mycelium completely covered the medium surface, and the flasks were prepared for samples’ exposure. After the first vibroacoustic measurement, all the specimens were sterilised using steam (10 min, 120 °C). All the samples were laid on the radial face (Figure 1b). For mechanical testing, 144 samples were exposed to fungal degradation (36 specimens/time period/fungal species). After 8 and 12 weeks of fungal exposure, all the specimens were removed from Kolle flasks, cleaned from superficial mycelium, and assessed with vibroacoustic techniques and static mechanical testing immediately afterwards. The MC, green density ($\rho_{w}$), and basic density ($\rho_{c}$) were calculated from sample dimensions and dry/wet (before and after degradation) masses.

### 2.3. Physical and Mechanical Measurements

A detailed description of the physical and mechanical measurements is presented by Cristini et al. [28].

#### 2.3.1. Vibroacoustic Measurements

The frequency resonance technique was used to determine the dynamic bending modulus of elasticity in the longitudinal–tangential direction of load (MOED). Mallet strikes on the top side caused oscillations. Vibrations of longitudinal–tangential bending modes were sensed using Doppler’s laser vibrometer. The records of vibrations were transformed from the time domain to the frequency domain using the fast Fourier transform (FFT) processed using MATLAB^®^ (The MathWorks, Inc., Natick, MA, USA). For comparison, 30 reference intact specimens (including the reference ones used for CT scanning) were also tested. All the reference samples went through the same impregnation process as all the other samples before inoculation (MC= 60 ± 10%).

#### 2.3.2. Static Mechanical Testing

Static three-point bending tests in a tangential direction were carried out on a universal testing machine. All the 30 reference specimens used for the vibroacoustic measurements were also mechanically tested. The modulus of rupture (MOR) was calculated as the bending stress in the tangential direction at the highest applied force. The static bending moduli of elasticity in the tangential direction (MOE) were established using a least-square method, fitting the data from the zone of linear elastic behaviour from the stress–strain diagram of each sample. The data were processed using MATLAB^®^. After testing, the ML for all the bending samples was calculated as for the mass-loss specimens.

#### 2.3.3. Computed Tomography Assessment

After static mechanical testing, all the samples were stored in a climatised room (20 °C, 65% relative humidity), together with 15 intact specimens, until reaching steady conditions. Afterwards, all the samples were scanned with a computed tomography (CT) system. The cross-sections of each sample were afterwards cropped in MATLAB^®^. The longitudinal density distribution of each specimen was obtained by importing all the cross-sections into MATLAB^®^ as numerical matrixes, and the mean value was calculated for each of them. The influence of the cracks produced during static testing on the density distribution was removed by replacing the crack zone with the values computed through a linear regression between the first intact cross-sections before and after the crack. The density derived from CT scanning ($\rho_{CT}$) was calculated for each specimen as the mean of the numerical vector used for the longitudinal density distribution.

### 2.4. Scanning Electron Microscopy

A scanning electron microscope (SEM) was used to conduct microscopy analysis. Small samples (4 × 4 × 4 mm) were sectioned from 12 different specimens. For mass-loss assessment, one sample was chosen from each measured period (2, 4, 6, and 8 weeks) for each species. The samples were chosen based on whether their mass loss was the closest to the average one for each sample group. For mechanical testing, one sample was chosen from each measured period (8 and 12 weeks) for each species. The latter were chosen according to their mass loss (as close to the average for each group) and depending on whether they had the most homogenous density distribution observed in the CT scans. All the small samples were prepared from the centre of each selected specimen. Sample preparation and microscopy setup are described in detail by Cristini et al. [28].

### 2.5. Statistical Analysis

The relationships between the measured parameters were statistically investigated in MATLAB^®^ (Spearman’s correlation coefficient (Sc) at α= 0.05, coefficient of variation (CV), and linear regression). Due to the higher variability of the measured data (the natural variability of wood material and the high variability caused by fungal deterioration), the ANOVA significance level (α) was set at 0.001, which is commonly used in wood science [34].

## 3. Results and Discussion

### 3.1. ML Comparison among Different Fungal Strains

**Figure 2:**
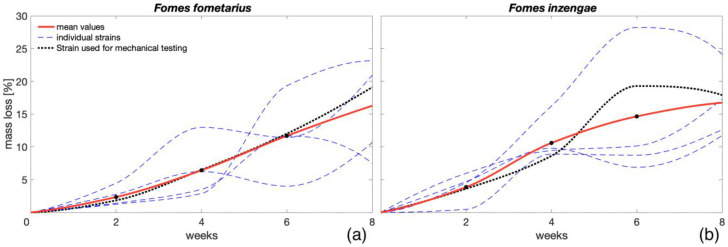
Average mass losses measured every two weeks of wood specimens exposed to different *F. fomentarius* (**a**) and *F. inzengae* (**b**) fungal strains.

According to the obtained results from the mass-loss experiment, there was no statistically significant difference in mass losses between the fungal species *F. fomentarius* and *F. inzengae* (p > 0.001), even when considering the high variability of the measured values among different strains of the same species (Figure 2). After eight weeks of fungal exposures, the average mass losses for both species were very similar (16.24% and 16.74% for *F. fomentarius* and *F. inzengae*, respectively).

Comparing the measured values to the results presented by Bari et al. [23], who measured the ML caused by *Pleurotus ostreatus* (Jacq.) P. Kumm. and *Trametes versicolor* (L.) Lloyd on beech wood after two months of fungal exposure (27% and 35%, respectively), both *Fomes* species showed relevantly lower average mass losses, even considering the fact that in both species, at least one strain showed an average ML of over 20% after 8 weeks (Figure 2). The CV of the measured mass losses among the different times of exposure varied from 43% to 84%. According to these results, both *Fomes* species can be considered less aggressive than the two fungal species presented in the latter research. Nevertheless, *Fomes* species are more oriented to pathogenicity than *T. versicolor* [16]; therefore, their enzymatic activity on wood in standing trees can differ from laboratory testing. The average MC values after 8 weeks of fungal exposure were also very close for both species (105% and 110% for *F. fomentarius* and *F. inzengae*, respectively), and no statistically significant difference between the two groups was found (p > 0.001). The obtained results are similar to the MC values presented by Bari et al. [23] for beech wood after 2 months of exposure to *P. ostreatus* (MC = 120%) but relevantly lower than the MC measured after the same exposure time for *T. versicolor* (MC = 210%). For all the mass-loss samples, a relevant correlation between ML and MC was observed (Sc = 0.74). This relationship was also found by Zelinka et al. [35], who revealed a linear increasing trend between the MC and ML in southern pine wood degraded due to its exposure to the brown-rot fungus *Rhodonia placenta* (Fr.) Niemelä, K.H. Larss. and Schigel. Bari et al. [23] also showed an increment in the MC with higher ML for both fungi *T. versicolor* and *P. ostreatus*. Nevertheless, while *T. versicolor* was found to have different MC depending on the colonised wood species, after 60 days of exposure, *P. ostreatus* tended to reach the same MC regardless of the exposed wood species and its ML.

**Figure 3:**
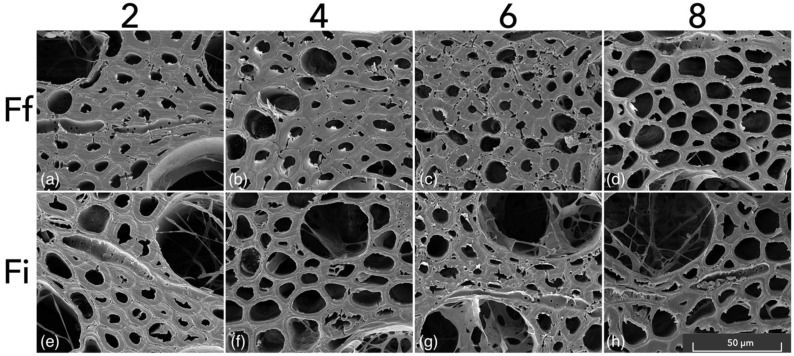
SEM scans of mass-loss-degraded samples exposed to *F. fomentarius* (**a**–**d**) and *F. inzengae* (**e**–**h**). Numbers refer to the inoculation period in weeks. Larger high-quality images are available in Supplementary Materials (Figures S1–S8).

As shown in Figure 3, the samples exposed to *F. fomentarius* and *F. inzengae* strains in Petri dishes showed decay patterns of both simultaneous white-rot (Figure 3d,f,h), deteriorating the cell wall eccentrically from the lumen and soft-rot decay type I [36] (Figure 3b,c,g), creating cavities in the S2 layer of the cell wall of fibre tracheids. Similar patterns were also found in the longitudinal sections of the examined specimens (Supplementary Figure S9). Different articles have already established the ability of different white-rot fungi to switch to type I soft rot, such as *Inonotus hispidus* [26], *Meripilus giganteus* [37], or *P. ostreatus* [23]. Skyba et al. [38] showed a shift to soft-rot behaviour by white-rot fungi due to the occlusion of the lumen of fibre tracheids of beech wood after a process of thermo-hygro-mechanical densification. In the specimens assessed with SEM, the cavities in the S2 layer of the cell wall were present mostly in fibre tracheids with thicker walls, often located close to and at the end of the annual growth. The penetration of wood elements by hyphae during decay mainly occurs through pit membranes or the cavities perpendicular to the cell wall [39,40,41]. Thus, the cavities in the S2 layer shown in Figure 3 should not be considered merely as penetration holes.

### 3.2. Physical and Mechanical Properties of Degraded Bending Samples

**Figure 4:**
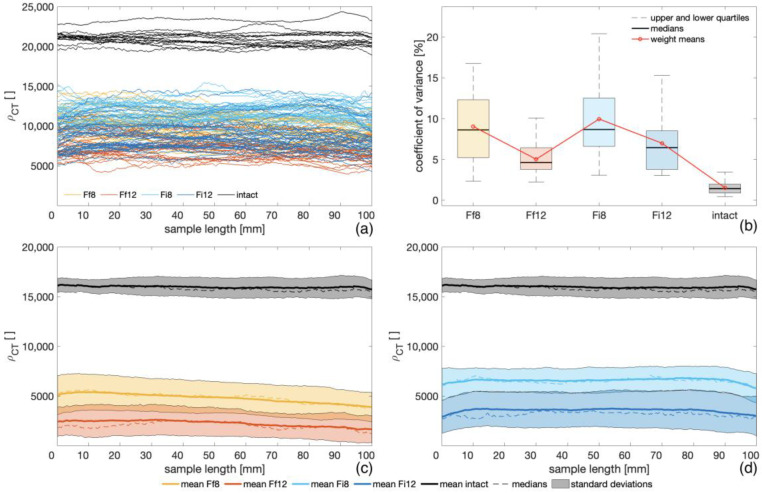
Density profile of all CT-scanned bending samples (**a**); variability in density distribution for each group of these samples (**b**); average values of density distribution with their standard deviations for intact samples and degraded specimens after 8 and 12 weeks of exposure to *F. fomentarius* (**c**) and *F. inzengae* (**d**) cultures.

According to the results obtained from the CT scanning of bending samples, the degraded samples had a significantly lower density than the intact ones (Figure 4a,c,d), and a statistically significant difference between the $\rho_{CT}$ measured for the degraded and intact samples was observed (p < 0.001). A significant difference was also observed for each fungal species between the samples exposed for 8 and 12 weeks. Comparing each exposure period for both fungal species, a statistically significant difference was observed for the 8-week group (p < 0.001), while no significant difference was observed between the two species after 12 weeks of fungal exposure (p > 0.001).

In comparison to the group of intact samples, the variability (coefficient of variance) in density distribution was found to be higher for all the degraded groups (Figure 4b). A statistically significant difference was observed between all the degraded samples and the intact ones (p < 0.001), while no significant difference was revealed between the two fungal species for each exposure time. Even if no statistically significant difference was found for *F. inzengae* between the two exposure times, as shown in Figure 4b, the variability in density distribution tended to decrease with a longer fungal exposure (higher level of degradation). Cristini et al. [28] presented the variability in density distribution for bending samples after 12 weeks of exposure to the soft-rot fungus *K. deusta* (average CV between 10% and 15%). The lower variability measured for the *Fomes* species can be explained by a higher level of degradation caused by both species in comparison to *K. deusta*, supporting the previous observation that with a more progressed deterioration, variability in density decreased. However, this difference in density distribution can be also caused by the different types of decay (white and soft rot).

A strong linear relationship was found between $\rho_{CT}$ and $\rho_{c}$ ($r^{2}=$ 0.99), proving the reliability of CT scanning for the assessment of the density of degraded samples. The presented results agree with the results proposed by Freyburger et al. [42], who presented an equivalently strong linear correlation ($r^{2}=$ 0.99) between CT scanning gravimetric methods for the density measurement of tropical wood specimens.

**Figure 5:**
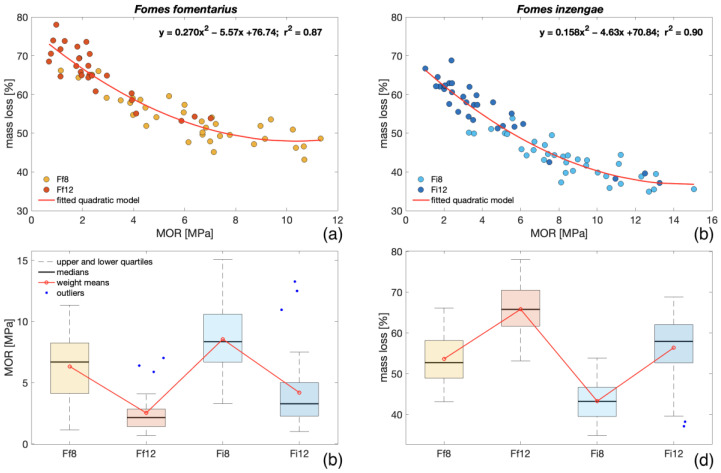
Relationships between ML and MOR for bending samples degraded by *F. fomentarius* (**a**) and *F. inzengae* (**b**), MOR (**c**), and ML (**d**) of all degraded samples exposed to both species for both exposure periods.

According to the results presented in Figure 5a,b, a strong quadratic relationship between the MOR and ML was observed. Nevertheless, based on ANOVA and multiple comparison tests, for the ML at each exposure period, a statistically significant difference between *F. fomentarius* and *F. inzengae* was found (p < 0.001), while this was not observed for the MOR (p > 0.001). For both parameters, *F. fomentarius* showed a slightly stronger degradation (Figure 5c,d), but in the case of the MOR, as stated before, these differences were not statistically relevant. In comparison to the ML measured for the samples exposed in Petri dishes, the ML in Kolle flasks after 8 weeks was more than 2 times higher (53% and 43% for *F. fomentarius* and *F. inzengae*, respectively). This fact can be explained by the results of Brischke et al. [43], who showed the impact of different specimens’ geometry on the ML after the same exposure time. Considering the different volumes of air contained in Petri dishes and Kolle flasks, another factor influencing the ML can be the concentration and availability of oxygen [44].

**Figure 6:**
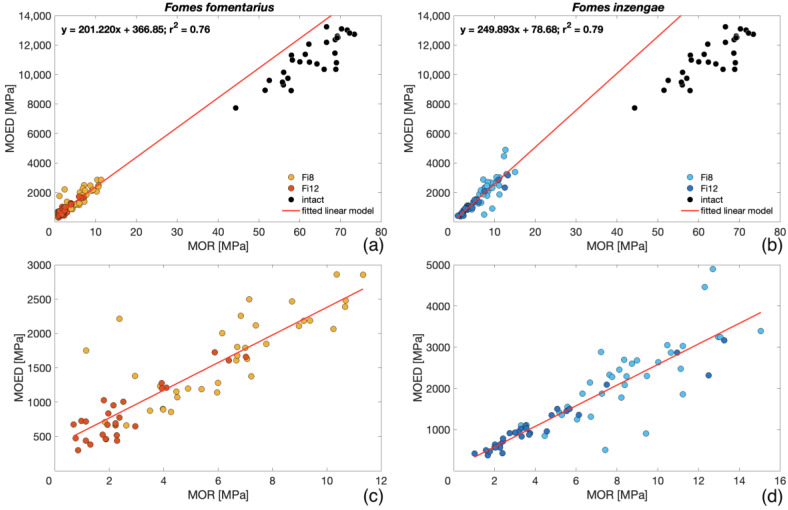
Linear relationship between MOR and MOED for bending specimens exposed to *F. fomentarius* (**a**) and *F. inzengae* (**b**) cultures and a closer representation without data from intact samples (**c**,**d**).

A strong linear relationship between the MOR and MOED was observed for specimens exposed to both *F. fomentarius* and *inzengae* ($Sc_{Ff}$ = 0.85; $Sc_{Fi}$ = 0.90) (Figure 6a,b). Nevertheless, in contrast to the results presented by Cristini et al. [28], where a linear model between MOR and MOED for the bending samples exposed to *K. deusta* was presented, the values shown in Figure 6a,b, which were calculated for the degraded wood, could not be applied to the intact samples. These results can be due to a more progressed degradation of the inoculated samples (higher mass loss) or by the different types of degradation (white rot and soft rot). Furthermore, considering the strong correlation between the static and dynamic bending moduli of elasticity (Sc = 0.93), it can be stated that vibroacoustic methods can be used for a reliable prediction of the static elastic properties of the wood degraded by both *Fomes* species, even at an advanced stage of degradation. In ANOVA and multiple comparison tests, no statistically significant difference was observed between the two species at each period of exposure (p > 0.001), corresponding to the results from the statistical analysis of MOR values. For both fungal species, a relevant negative correlation between MC and MOR was found ($Sc_{Ff}$ = −0.54; $Sc_{Fi}$ = −0.75). These results were attributed to the relationship between ML and MC ($Sc_{Ff}$ = −0.60; $Sc_{Fi}$ = −0.74), which was already observed for the mass-loss specimens exposed in Petri dishes. These relationships are also evident in the average values presented in Table 2. In comparison to the average values of the physical and mechanical properties measured for the reference intact samples, the degraded specimens showed a relevantly higher variability (Table 2) caused by fungal enzymatic activity.

**Figure 7:**
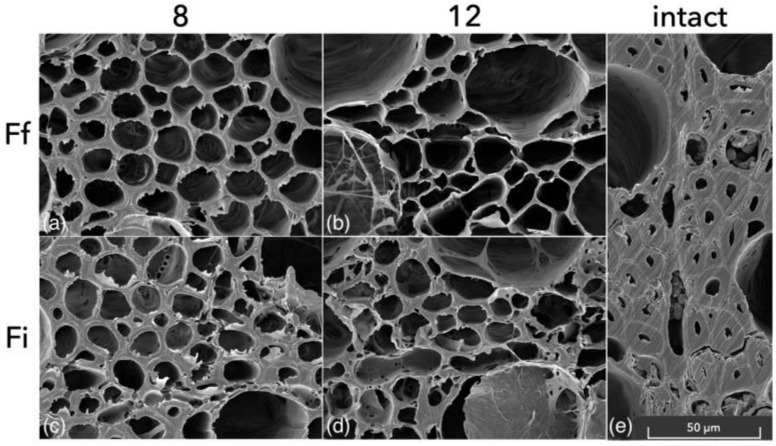
SEM scans of bending samples exposed to *F. fomentarius* (**a**,**b**) and *F. inzengae* (**c**,**d**) cultures; scan from intact bending sample (**e**). Numbers refer to the inoculation period in weeks. Larger high-quality images are available in Supplementary Materials (Figures S10–S13).

As shown in Figure 7, all the bending samples assessed with SEM showed patterns of simultaneous white rot for both species. In the more advanced stage (after 12 weeks of fungal exposure), it was possible to identify the typical final stage of white-rot degradation, where the compound middle lamella was also being deteriorated (Figure 7b,d). The advanced stage of decay, already observed after two months of fungal exposure, is even more evident if compared with the scan obtained from an intact sample from the reference group (Figure 7e).

In the bending samples selected for SEM, the signs of cavities in the S2 layer of the fibre tracheids, as shown for the mass-loss specimens (Figure 3). These results can be explained by an already too-advanced stage of degradation, where all the cavities were already connected causing a thinning of the cell wall. The more common presence of soft-rot patterns in the mass-loss samples could also be caused by the different environmental conditions between cultures in Petri dishes and Kolle flasks.

## 4. Conclusions

Considering the previously discussed results, the following conclusive statements can be presented:

- From the experiment conducted on different strains of *F. fomentarius* and *F. inzengae*, no relevant difference in ML and MC between the two species was found (p > 0.001). Both species showed similar trends and high variability among the different strains. With an increment in the ML, MC increased for both species (Sc = 0.74).
- The degraded bending samples showed higher heterogeneity of density distribution than the intact ones (p < 0.001). After 8 weeks of fungal exposure, a difference in densities was observed between the two species. Nevertheless, after 12 weeks, no relevant difference was found.
- A strong relationship between ML and MOR was found for both species (Figure 5). After both exposure periods, no relevant difference in the MOR between the two species was evident (p > 0.001).
- The higher ML of bending samples compared with the specimens exposed in Petri dishes was probably caused by the samples’ geometry and environmental conditions. The negative correlation between the MOR and MC for both species (Sc between −0.54 and −0.74) was caused by the increasing trend of MC with ML.
- For both species, the MOED and MOR showed a strong linear relationship (Figure 6). As for the MOR, no relevant difference between the two species after both exposure times was observed.
- According to our SEM assessment, both species showed the morphological patterns of simultaneous white rot and soft-rot type I.

Based on the relationships among the investigated material parameters in this study, there were no significant differences found in the mechanical and physical properties of beech wood degraded by *F. fomentarius* and *F. inzengae*. Even if these two species are considered distinct, their presence and impact on a colonised tree can be interpreted equally during the stability assessment of standing trees. The strong relationship found between the strength loss and MOED can be used to improve the nondestructive, device-supported methods used for tree stability assessment.

## Figures and Tables

**Table 1:** Fungal strains used for fungal exposure of mass-loss samples and original tree host species from which basidiomes were collected. Bold strains were used for the fungal exposure of samples for mechanical testing.

Species	*Fomes fomentarius*					*Fomes inzengae*				
Strain	Ff1	Ff2	Ff3	Ff4	Ff5	Fi1	Fi2	Fi3	Fi4	Fi5
Host	*Fagus sylvatica*	*Betula* sp.	*Betula* sp.	*Fagus sylvatica*	*Fagus sylvatica*	*Fagus sylvatica*	*Quercus petraea*	*Fagus sylvatica*	*Alnus glutinosa*	*Tilia cordata*
Accession N.*	OQ474927	OQ474923	OQ474926	OQ474928	OQ474930	OQ474914	OQ474913	OQ474917	OQ474918	OQ474921

* GenBank accession numbers of the ITS sequences of isolates deposited in the NCBI database.

**Table 2:** Summary of average values for physical and mechanical properties with their coefficients of variation for both species at each exposure period and reference intact specimens.

	*MOR* (MPa)	*MOED* (MPa)	$\rho_{w}$ (kg/m^3^)	$\rho_{C}$ (kg/m^3^)	*MC* (%)	*ML* (%)
Ff 8	6.3 (42.9%)	1 685 (36.8%)	681.7 (15.9%)	265.8 (12.6%)	161.3 (36.4%)	53.6 (11.2%)
Ff 12	2.5 (66.2%)	817 (49.1%)	561.2 (27.3%)	196.4 (19.5%)	203.4 (59.2%)	65.8 (10.2%)
Fi 8	8.5 (33%)	2 250 (43.1%)	941.7 (19%)	326.5 (8.9%)	190.5 (31.3%)	43.3 (11.6%)
Fi 12	4.2 (73.5%)	1 088 (63.7%)	1017 (8.73%)	252.7 (17.9%)	312.2 (21.8%)	56.4 (14.2%)
Ref	63 (11.5%)	11 102 (13.3%)	925.9 (3.1%)	558.9 (3.5%)	65.8 (9.8%)	

MOR—modulus of rupture, MOED—dynamic modulus of elasticity in bending, $\rho_{w}$—green density, $\rho_{C}$—basic density, MC—moisture content, and ML—mass loss.

## Data Availability

The data presented in this study are available on request from the corresponding author.

## Supplementary Materials

The following supporting information can be downloaded at: https://www.mdpi.com/article/10.3390/microorganisms11030679/s1, Figure S1: SEM scans of mass-loss-degraded sample exposed to *F. fomentarius* for 2 weeks in Petri dish—cross-section; Figure S2: SEM scans of mass-loss-degraded sample exposed to *F. fomentarius* for 4 weeks in Petri dish—cross-section; Figure S3: SEM scans of mass-loss-degraded sample exposed to *F. fomentarius* for 6 weeks in Petri dish—cross-section; Figure S4: SEM scans of mass-loss-degraded sample exposed to *F. fomentarius* for 6 weeks in Petri dish—cross-section; Figure S5: SEM scans of mass-loss-degraded sample exposed to *F. inzengae* for 2 weeks in Petri dish—cross-section; Figure S6: SEM scans of mass-loss-degraded sample exposed to *F. inzengae* for 4 weeks in Petri dish—cross-section; Figure S7: SEM scans of mass-loss-degraded sample exposed to *F. inzengae* for 6 weeks in Petri dish—cross-section; Figure S8: SEM scans of mass-loss-degraded sample exposed to *F. inzengae* for 8 weeks in Petri dish—cross-section; Figure S9: SEM scans of mass-loss-degraded sample exposed to *F. inzengae* for 6 weeks in Petri dish—longitudinal section; Figure S10: SEM scans of mass-loss-degraded sample exposed to *F. fomentarius* for 8 weeks in Kolle flask—cross-section; Figure S11: SEM scans of mass-loss-degraded sample exposed to *F. fomentarius* for 12 weeks in Kolle flask—cross-section; Figure S12: SEM scans of mass-loss-degraded sample exposed to *F. inzengae* for 8 weeks in Kolle flask—cross-section; Figure S13: SEM scans of mass-loss-degraded sample exposed to *F. inzengae* for 12 weeks in Kolle flask—cross-section.
