# Supplementary material for: Fomes fomentarius and F. inzengae—A Comparison of Their Decay Patterns on Beech Wood
Source: Microorganisms. 2023 Mar 7;11(3):679. doi: 10.3390/microorganisms11030679 (PMC10056366; doi:10.3390/microorganisms11030679)
Supplement: Supplementary file 1 [file microorganisms-11-00679-s001.zip › microorganisms-2196608-supplementary.pdf]

Supplementary material

Fomes fomentarius and *F. inzengae* – a comparison of their decay patterns on beech wood

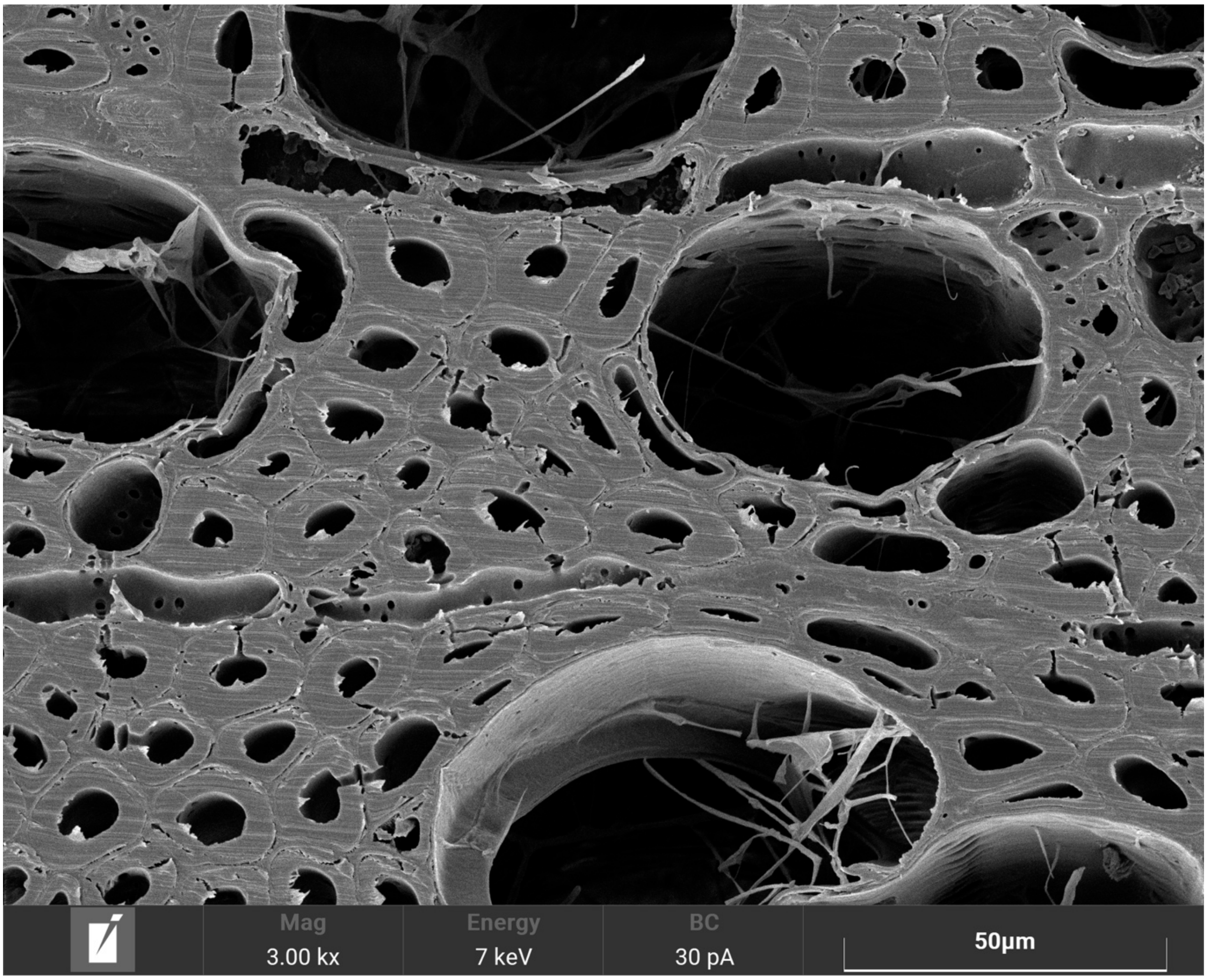

Figure S1. SEM scans of mass-loss-degraded sample exposed to *F. fomentarius* for 2 weeks in Petri dish – cross-section.

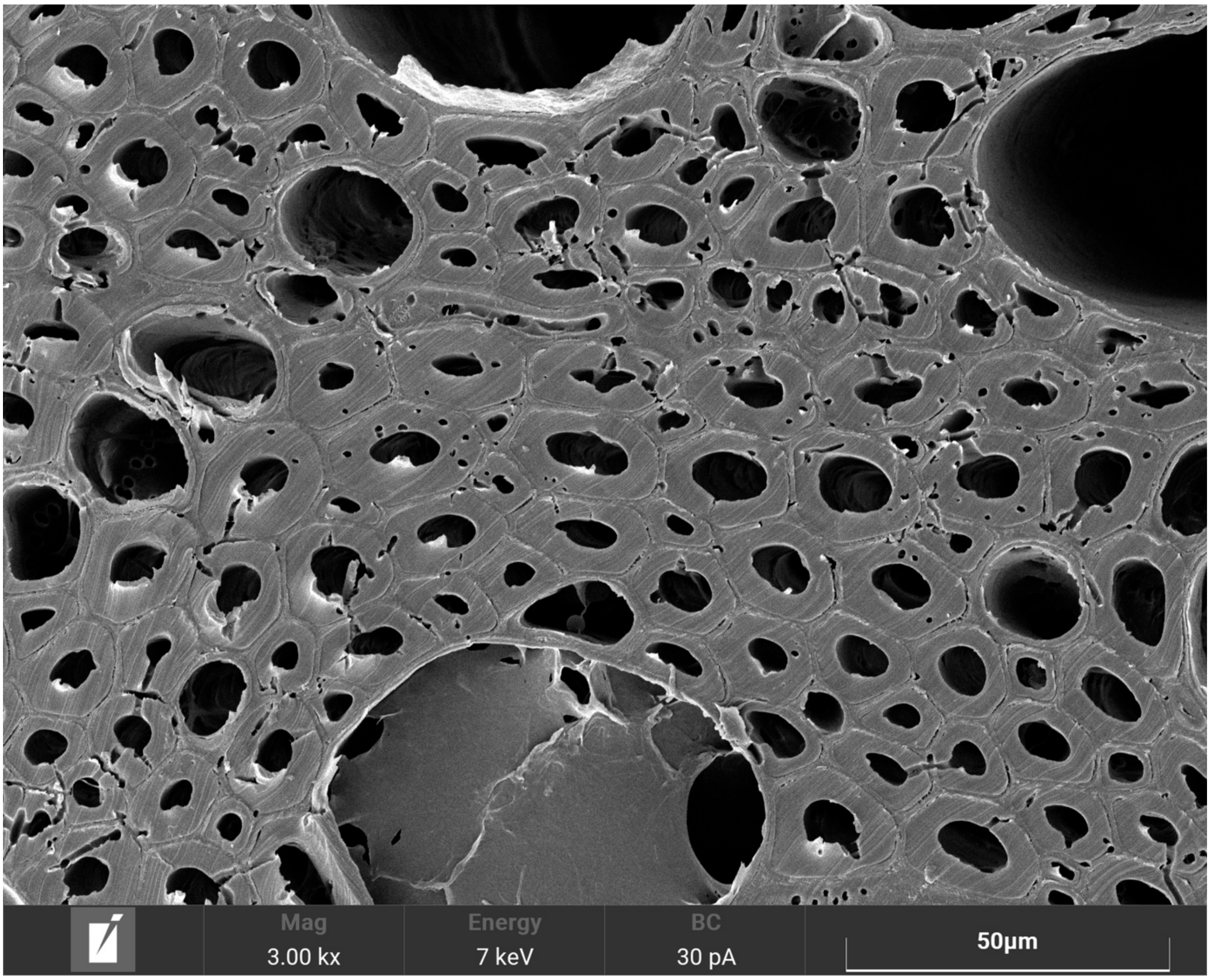

Figure S2. SEM scans of mass-loss-degraded sample exposed to *F. fomentarius* for 4 weeks in Petri dish – cross-section.

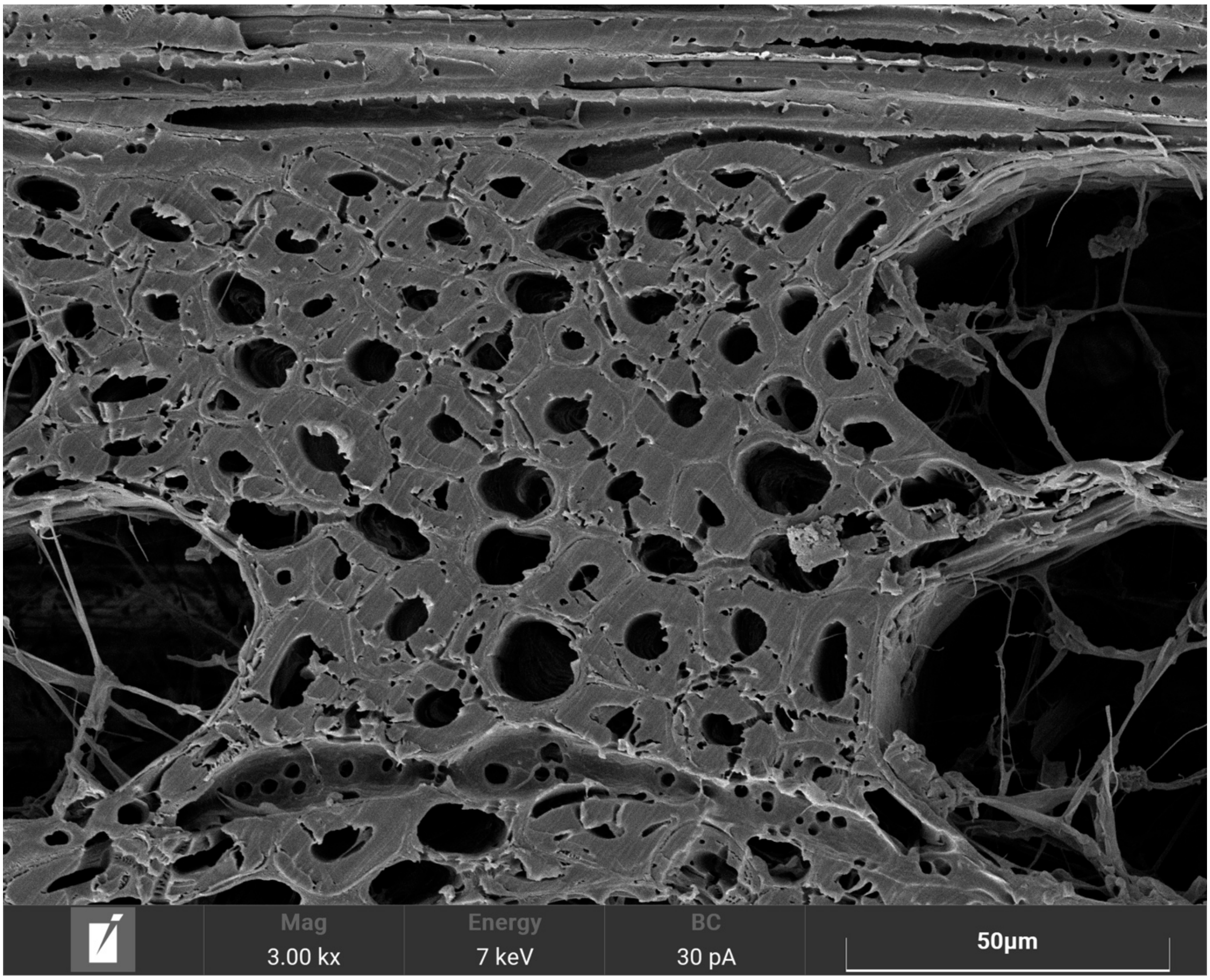

Figure S3. SEM scans of mass-loss-degraded sample exposed to *F. fomentarius* for 6 weeks in Petri dish – cross-section.

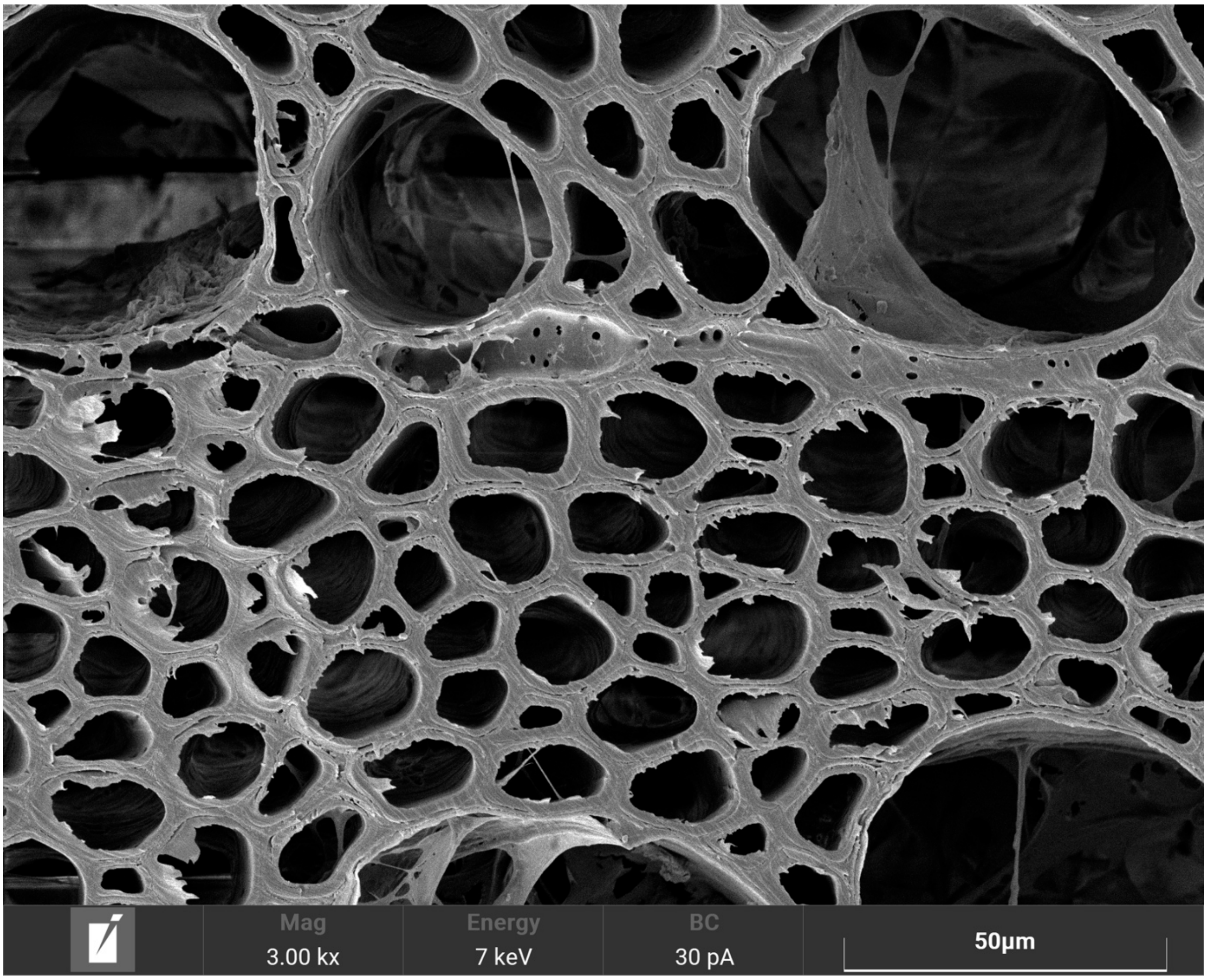

Figure S4. SEM scans of mass-loss-degraded sample exposed to *F. fomentarius* for 6 weeks in Petri dish – cross-section.

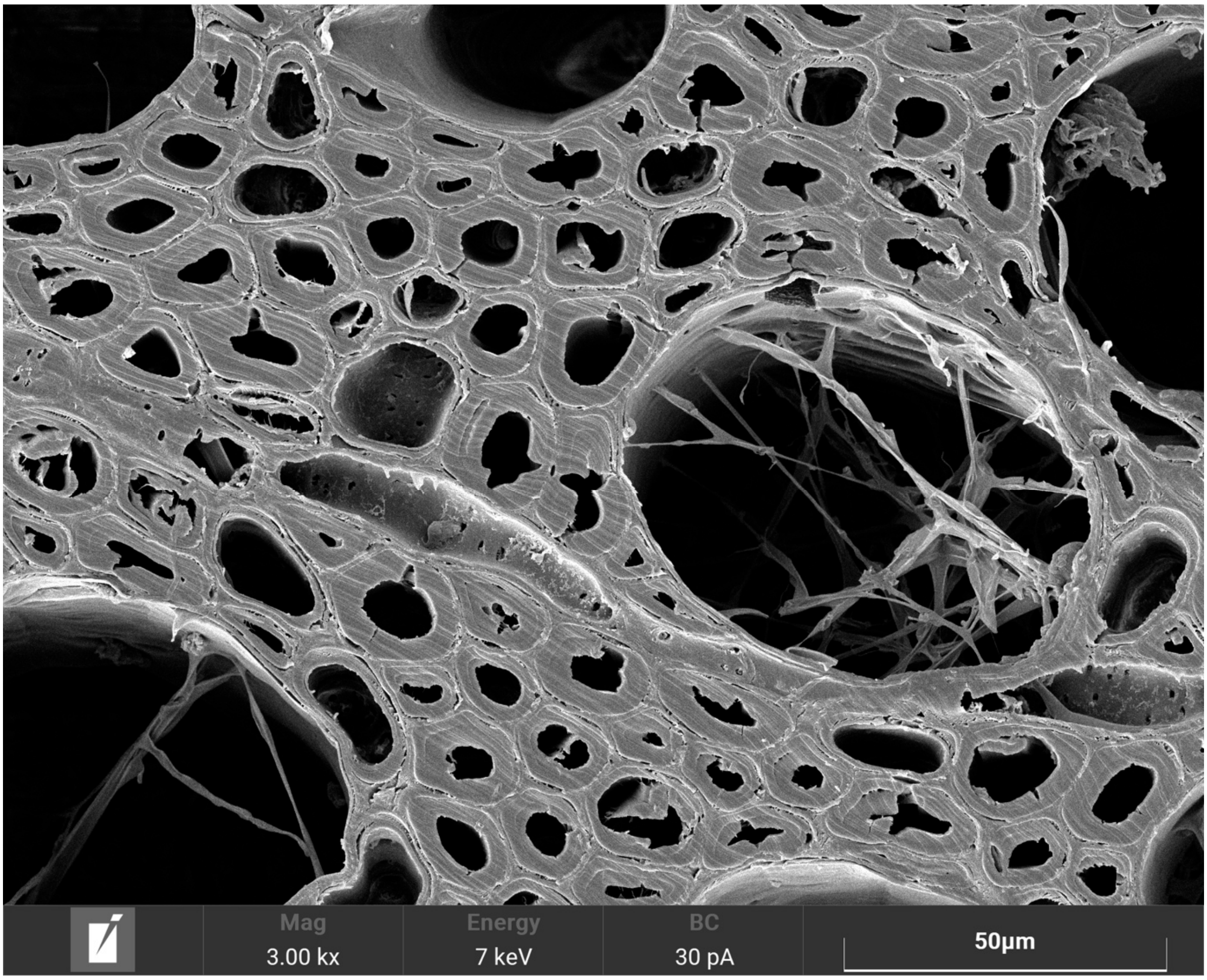

Figure S5. SEM scans of mass-loss-degraded sample exposed to *F. inzengae* for 2 weeks in Petri dish – cross-section.

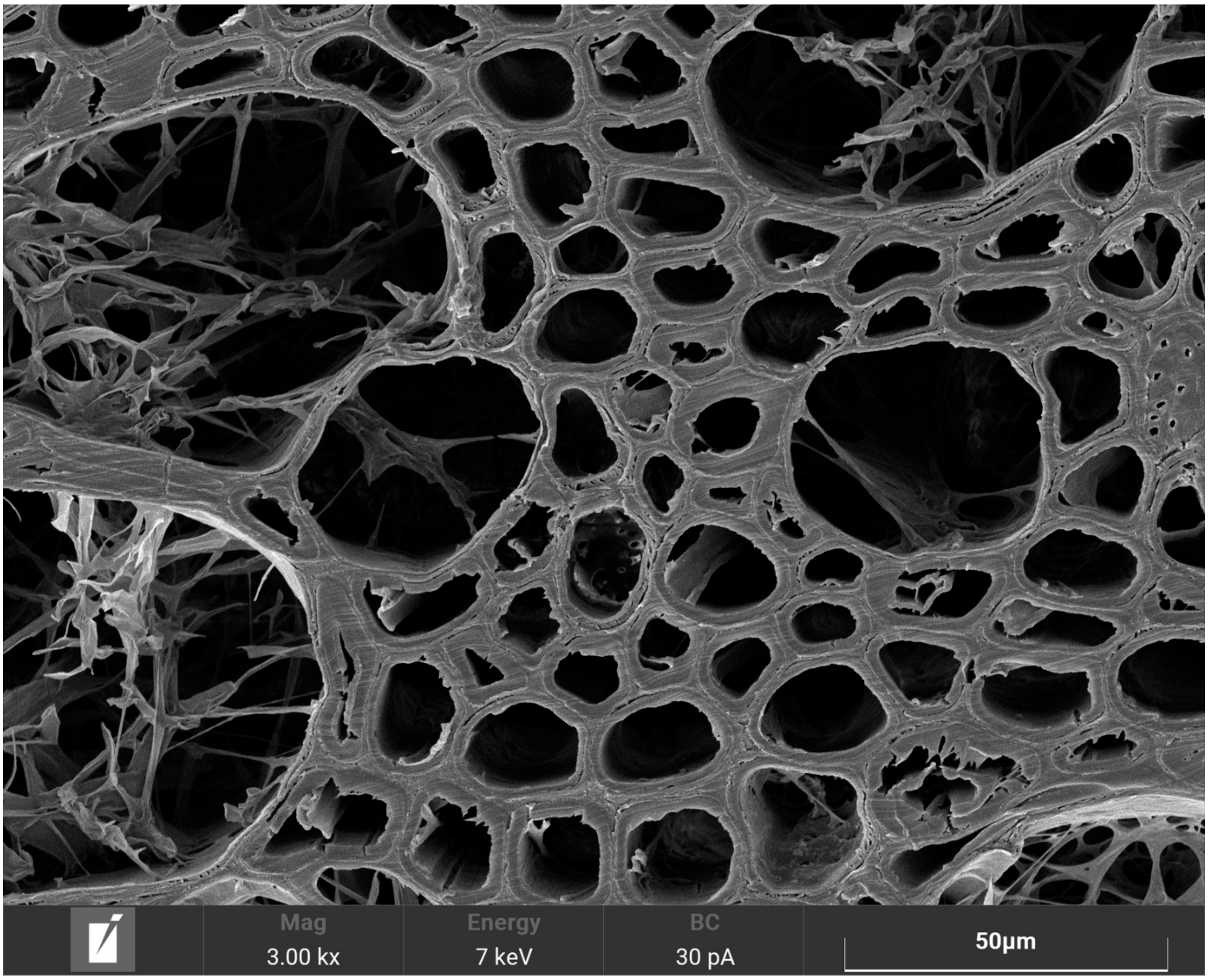

Figure S6. SEM scans of mass-loss-degraded sample exposed to *F. inzengae* for 4 weeks in Petri dish – cross-section.

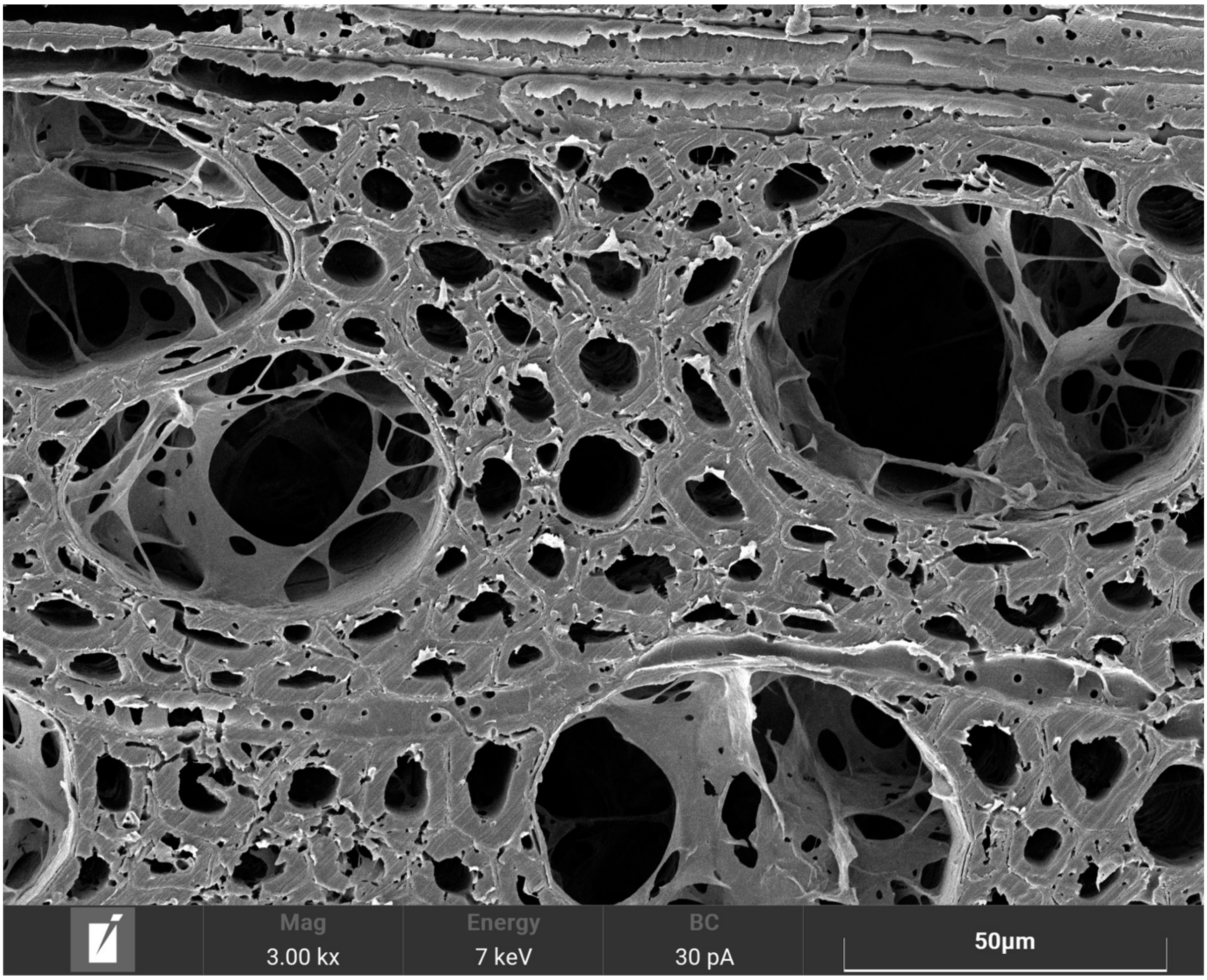

Figure S7. SEM scans of mass-loss-degraded sample exposed to *F. inzengae* for 6 weeks in Petri dish – cross-section.

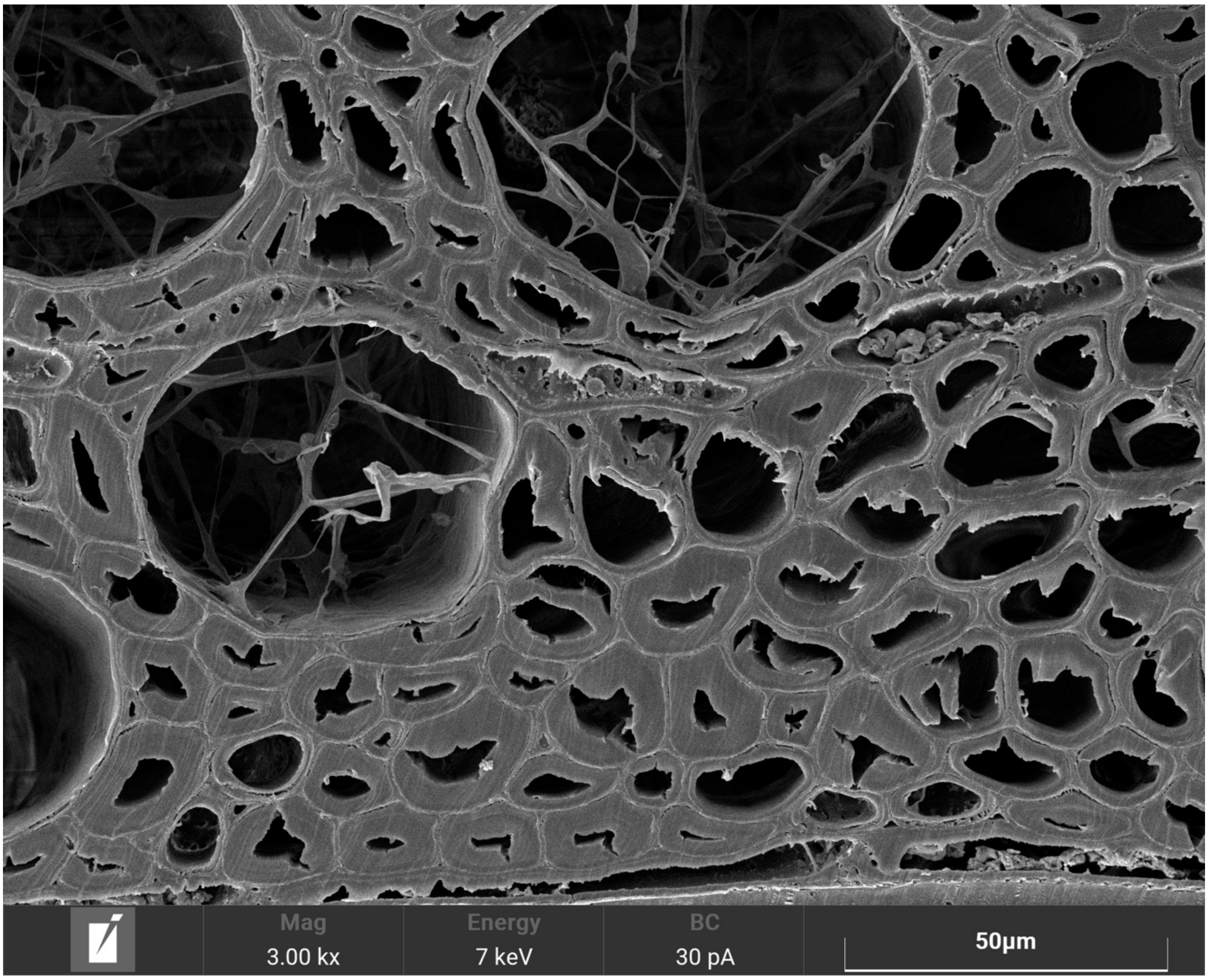

Figure S8. SEM scans of mass-loss-degraded sample exposed to *F. inzengae* for 8 weeks in Petri dish – cross-section.

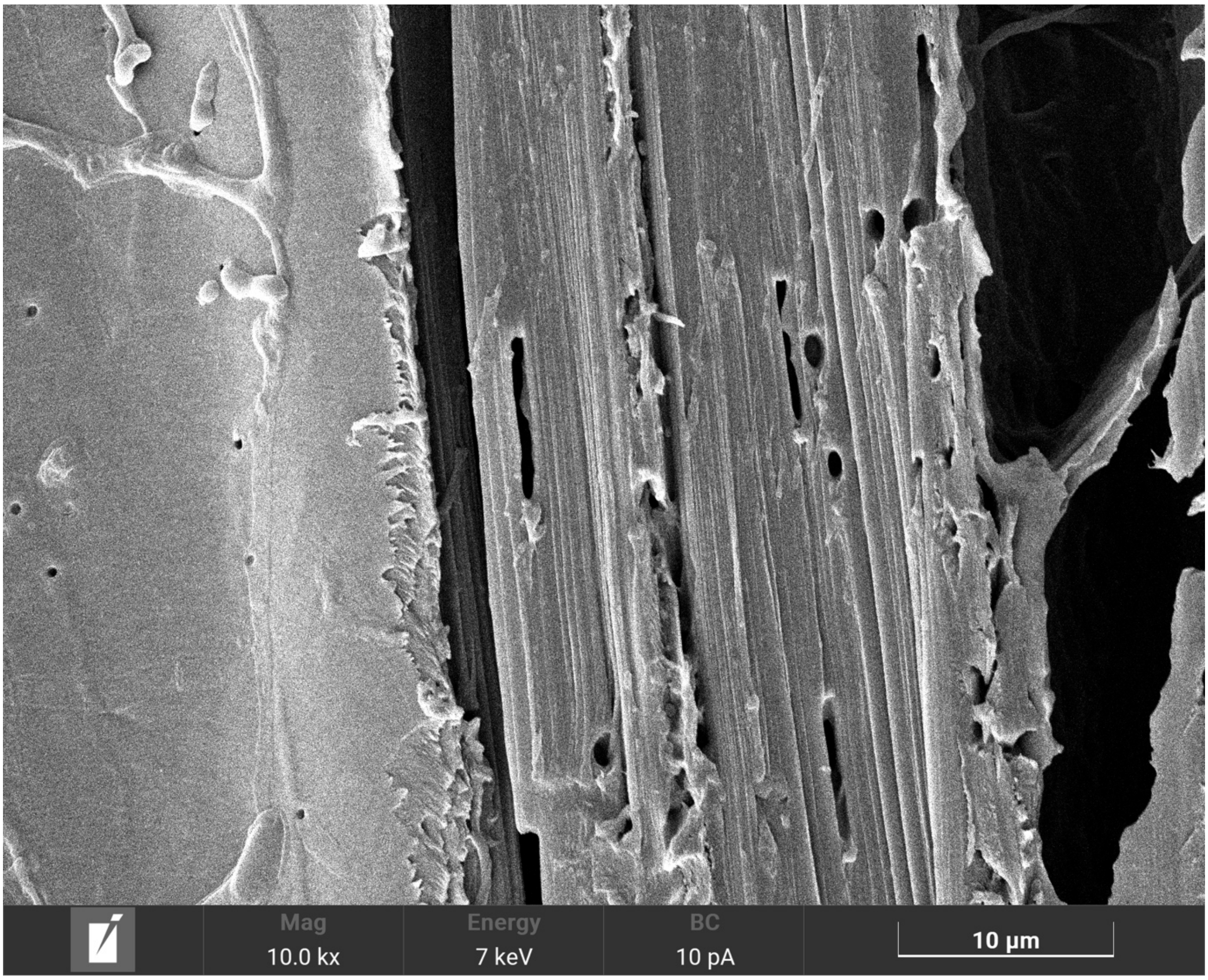

Figure S9. SEM scans of mass-loss-degraded sample exposed to *F. fomentarius* for 6 weeks in Petri dish – longitudinal section.

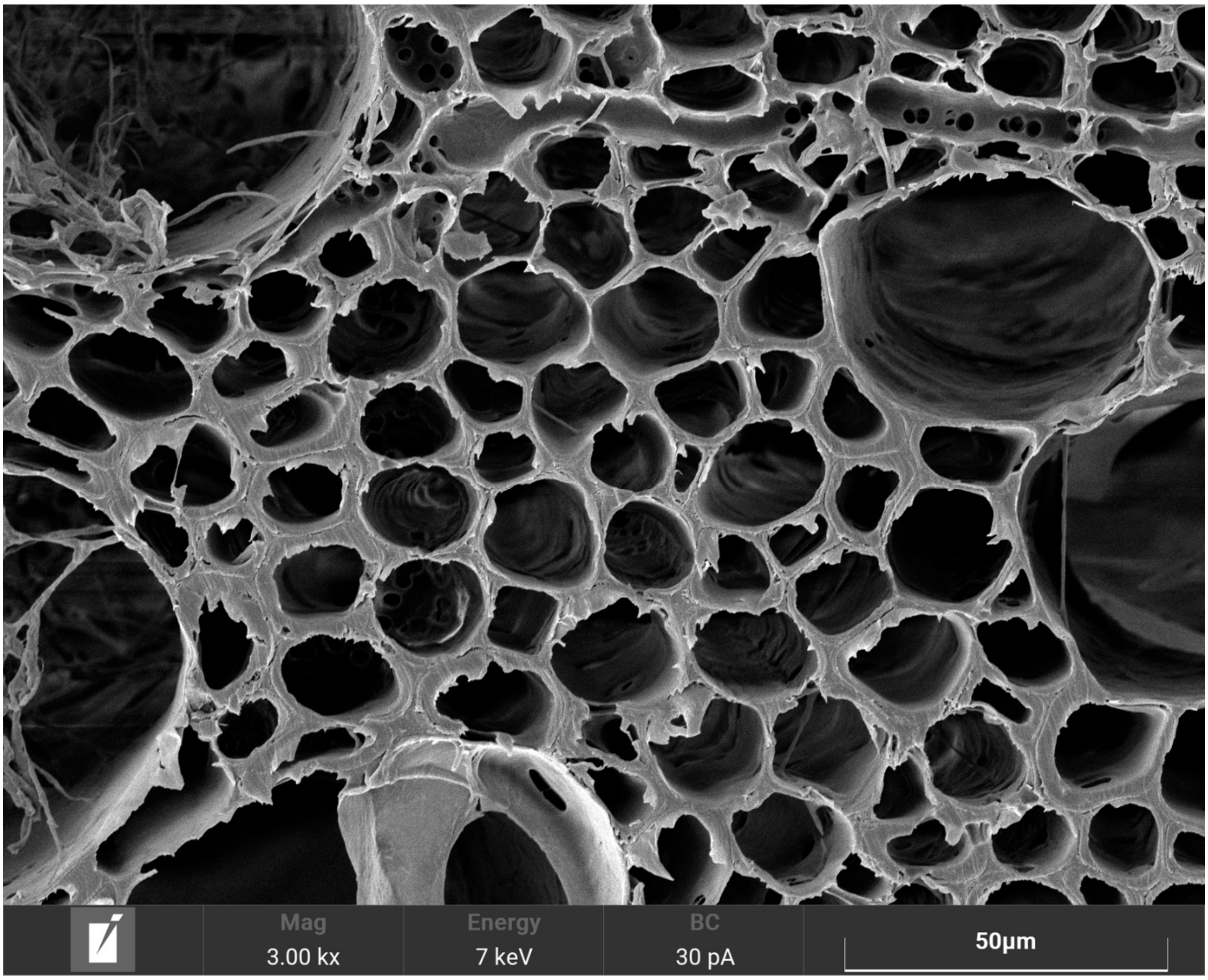

Figure S10. SEM scans of mass-loss-degraded sample exposed to *F. fomentarius* for 8 weeks in Kolle flask – cross-section.

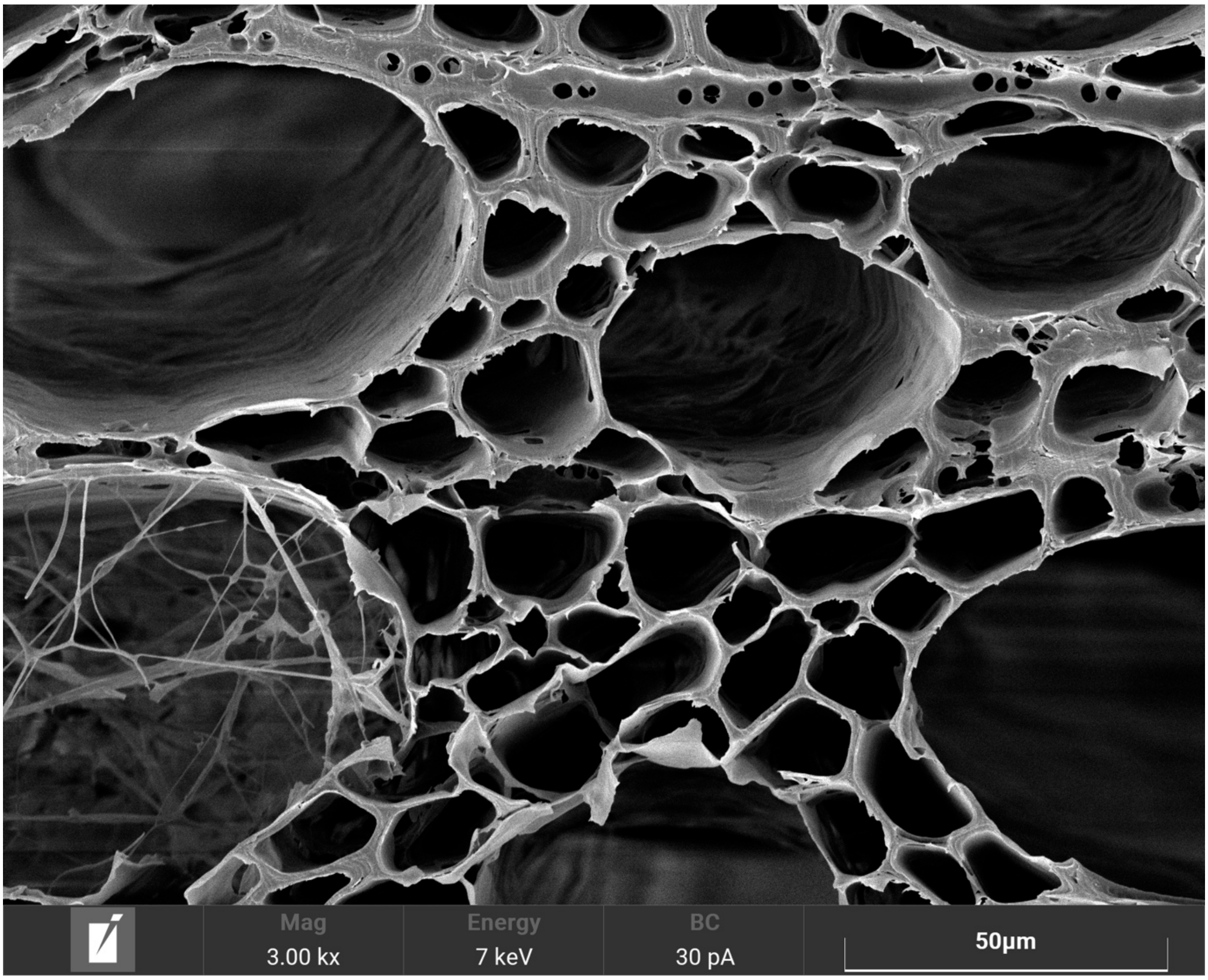

Figure S11. SEM scans of mass-loss-degraded sample exposed to *F. fomentarius* for 12 weeks in Kolle flask – cross-section.

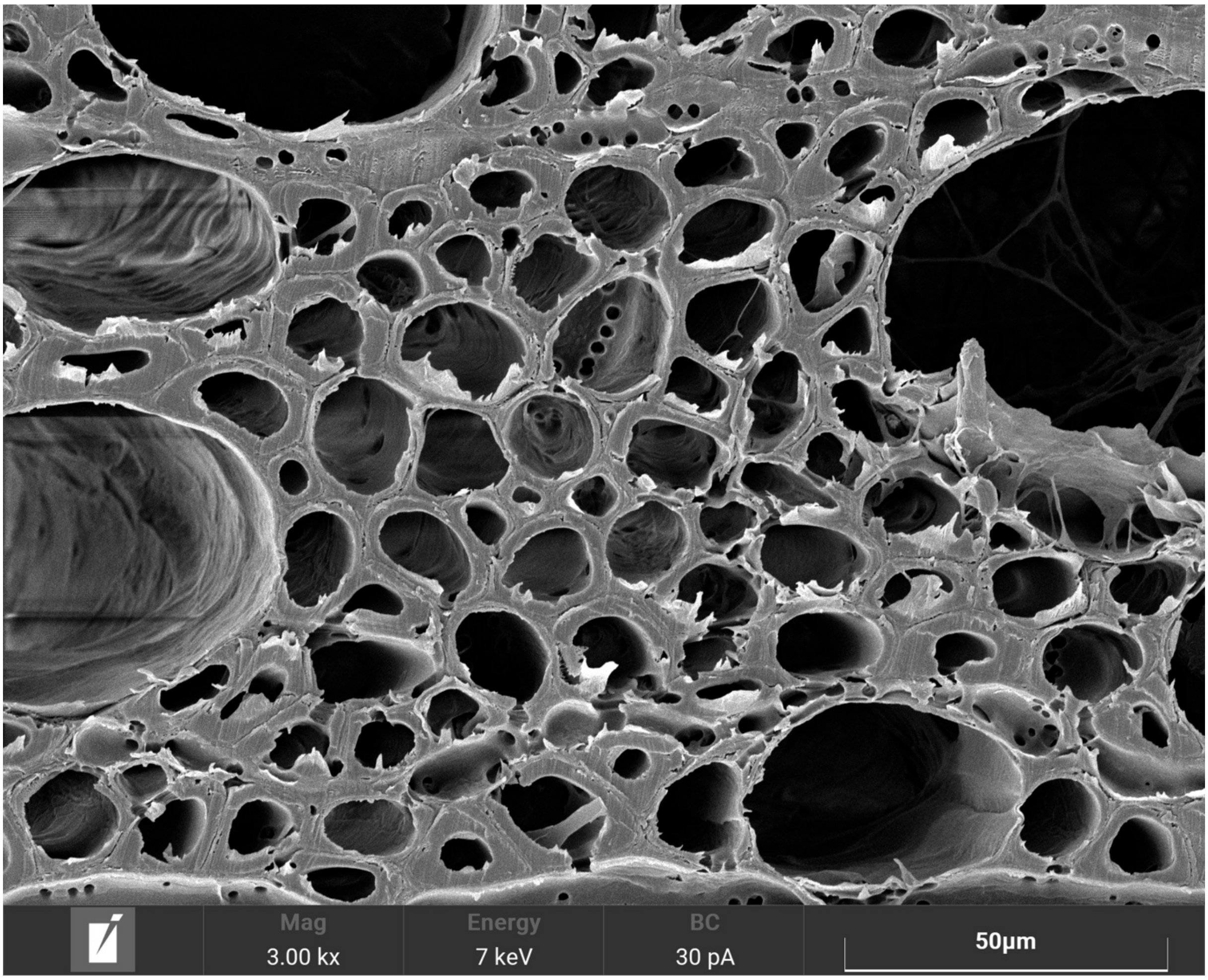

Figure S12. SEM scans of mass-loss-degraded sample exposed to *F. inzengae* for 8 weeks in Kolle flask – cross-section.

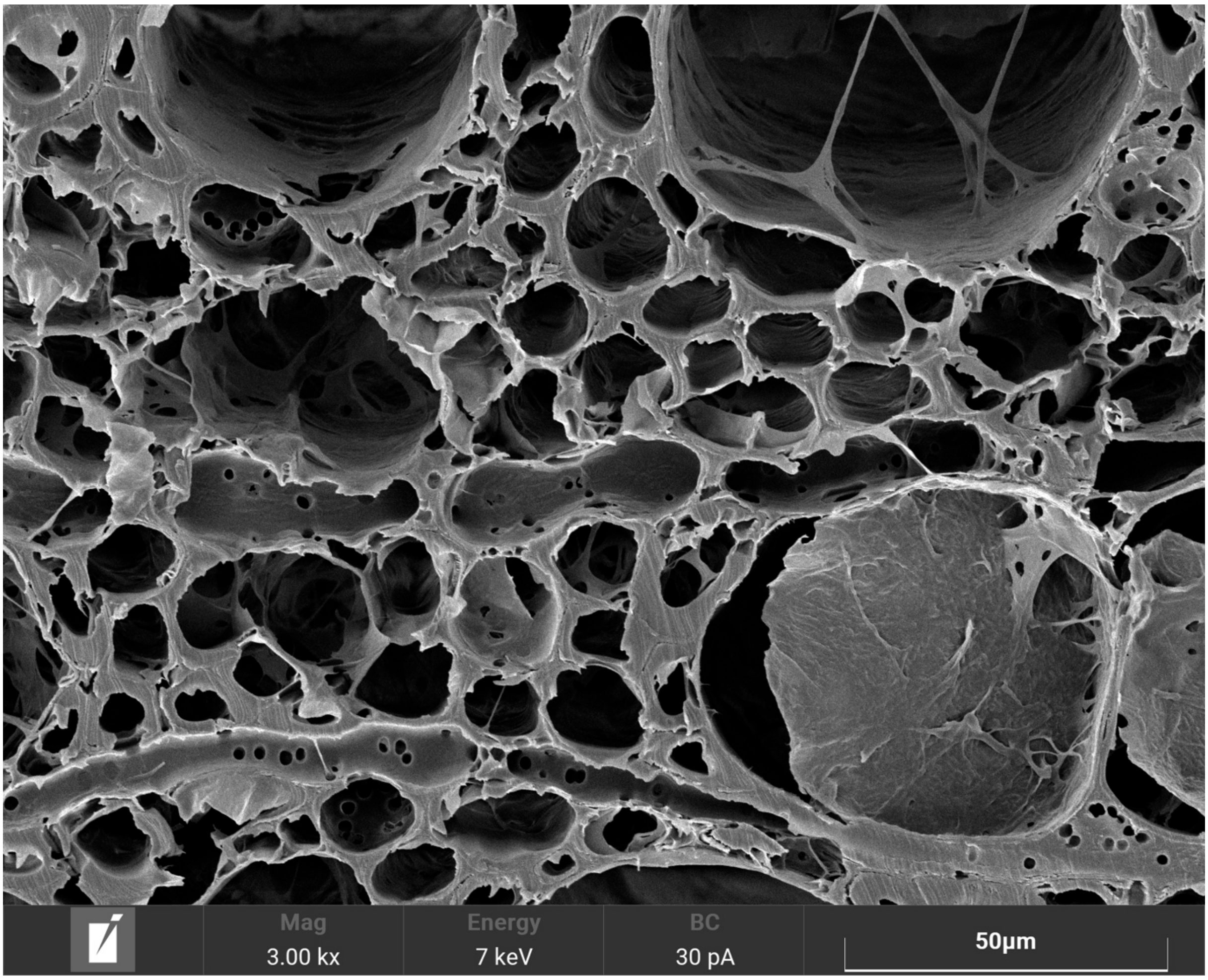

Figure S13. SEM scans of mass-loss degraded sample exposed to *F. inzengae* for 8 weeks in Kolle flask – cross-section.
